# The Mitochondrial Protein Translocation Motor: Structural Conservation between the Human and Yeast Tim14/Pam18-Tim16/Pam16 co-Chaperones

**DOI:** 10.3390/ijms10052041

**Published:** 2009-05-06

**Authors:** Shira Elsner, Dana Simian, Ohad Iosefson, Milit Marom, Abdussalam Azem

**Affiliations:** Department of Biochemistry, George S. Wise Faculty of Life Sciences, Tel Aviv University, Tel Aviv 69978, Israel; E-Mails: shiraelsner@gmail.com (S.E.); simianda@post.tau.ac.il (D.S.); iosefson@post.tau.ac.il (O.I.); militm@yahoo.com (M.M.)

**Keywords:** Tim14 (also known as Pam18), Tim16 (also known as Pam16), mtHsp70, translocation motor

## Abstract

Most of our knowledge regarding the process of protein import into mitochondria has come from research employing *Saccharomyces cerevisiae* as a model system. Recently, several mammalian homologues of the mitochondrial motor proteins were identified. Of particular interest for us is the human Tim14/Pam18-Tim16/Pam16 complex. We chose a structural approach in order to examine the evolutionary conservation between yeast Tim14/Pam18-Tim16/Pam16 proteins and their human homologues. For this purpose, we examined the structural properties of the purified human proteins and their interaction with their yeast homologues, *in vitro*. Our results show that the soluble domains of the human Tim14/Pam18 and Tim16/Pam16 proteins interact with their yeast counterparts, forming heterodimeric complexes and that these complexes interact with yeast mtHsp70.

## 1. Introduction

Mitochondria are vital organelles for eukaryotes because they serve as a site for many essential processes such as respiration, lipid metabolism, heme metabolism, synthesis of metabolites and metal homeostasis. Moreover, in higher eukaryotes, mitochondria participate in calcium signaling and in mediation of apoptosis. It is estimated that ~800–1,000 proteins are involved in these and other mitochondrial functions. However, only a very small fraction of the mitochondrial proteins is actually produced *in situ* (eight in *S. cerevisiae*). The rest are encoded by nuclear genes, synthesized in the cytosol and then delivered to one of the four mitochondrial compartments: the outer membrane, inner membrane, the intermembrane space and the matrix. Consequently, functional import systems for nuclear encoded proteins are indispensable for the biogenesis of mitochondria and accordingly for the viability of eukaryotic cells. The import of nuclear-encoded proteins into the mitochondria is a multistep process mediated by the coordinated action of translocation machineries localized in both the outer and inner mitochondrial membranes [1–3].

In the outer membrane, the multimeric TOM complex serves as both a receptor for recognition of mitochondrial precursor proteins and a main portal of protein entry into mitochondria [1–4]. The TOM complex is composed of the primary receptors, Tom20 and Tom70, and the subunits Tom40, Tom22, Tom7, Tom6, and Tom5 that together form the stable core of the complex. Tom40 forms the protein-conducting channel, providing a route for precursor proteins to cross the outer membrane. On their way to the matrix, proteins that contain cleavable amino-terminal targeting signals are transferred from the TOM complex to the TIM23 preprotein translocase. This complex is composed of a core of two multispanning integral inner membrane proteins Tim17 and Tim23. The latter forms a channel which allows proteins to integrate into, or to cross, the inner membrane. A third protein, Tim50, seems to serve as a sorting receptor in the mitochondrial intermembrane space and maintains the permeability barrier that is formed by Tim23 [5].

The final steps of translocation across the inner membrane are mediated by the mitochondrial translocation motor (Presequence translocase-Associated protein import Motor, PAM). The translocation motor itself is a multisubunit complex, which contains the ATP-hydrolyzing 70 kDa heat-shock protein, mtHsp70, as its central component. Precursor proteins emerging from the matrix side of the TIM23 channel bind to mtHsp70, with whose help the protein unfolds and completes its translocation into the mitochondrial matrix [2,6–9]. In order to mediate protein import, mtHsp70 must anchor to the TIM23 import channel at certain stages of the import process. This is accomplished by another component of the translocation motor, Tim44, a peripheral membrane protein that binds simultaneously to mtHsp70 and the TIM23 complex [10–13]. MtHsp70 undergoes conformational changes that are controlled by ATP hydrolysis upon binding, unfolding and release of precursor proteins.

Additional components of the motor are suggested to play a regulatory role in either the function or stability of the motor. These include accessory proteins that directly regulate the function of mtHsp70 [7]. For example, Mge1 acts as a nucleotide-exchange factor and promotes the release of imported precursor proteins from mtHsp70. J-domain containing proteins, DnaJs, usually enhance the ATPase activity of Hsp70 chaperones. Such enhancement of the ATPase activity is required for promoting the tight binding of unfolded substrate proteins to the peptide-binding pocket of mtHsp70. The latter role is played by the membrane-bound J-domain-containing protein named Tim14/Pam18 [11,14,15]. Biochemical and in-organelle studies has shown that Tim14/Pam18 forms a stable complex with another protein, Tim16/Pam18 [16–18] which acts as an antagonist to the Tim14/Pam18 ATPase enhancement [16,19]. High resolution structure of the complex has been published recently and provided valuable mechanistic insight into the function of the yeast Tim14/Pam18-Tim16/Pam16 heterdimer [20].

Homologues of yeast Tim14/Pam18 and Tim16/Pam16 were also identified in humans (DNAJC19 and Magmas respectively). DNAJC19 and Magmas are of great interest since they are associated with several human disorders. A novel autosomal recessive disorder called “DCMA syndrome” has already been associated with a mutation in the DNAJC19 protein [21], while Magmas is suspected to be involved in increased rates of anaerobic metabolism, resistance to apoptosis and altered growth-factor sensitivity, characteristic of cancer cells [22–25]. In this study, we used recombinantly purified proteins to investigate the ability of human Tim14/Pam18 and Tim16/Pam16 to form hetero-oligomers with each other and with their yeast homologues. Our results suggest that there is structural conservation between the yeast and human homologues.

## 2. Results and Discussion

The aim of this work was to study *in vitro*, for the first time, the structural properties of two human disease-associated proteins, Tim14/Pam18 and Tim16/Pam16 (also named DNAJC19 and Magmas, respectively). It is noteworthy that, while the yeast proteins have been studied extensively both *in vivo* and *in vitro*, little has been done to study the human proteins. Even complex formation between human Tim14/Pam18 and Tim16/Pam16 has never been demonstrated. In particular, we wanted to examine the following aspects. i) Do human Tim14/Pam18 and Tim16/Pam16, similar to their yeast homologues, interact to form a stable complex? ii) Which forces stabilizes the human Tim14/Tim16 complex? iii) Do the human Tim14/Pam18 and Tim16/Pam16 proteins form stable complexes with their yeast counterparts? Answers to these questions will provide important information on the evolutionary conservation of these complexes.

### 2.1. Purification of a Tim14/Pam18 - Tim16/Pam16 complex

**Figure 1.**
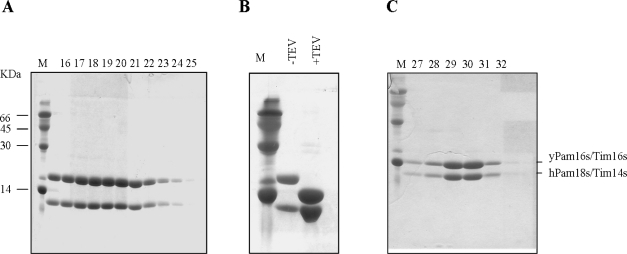
SDS–PAGE analysis of the purified recombinant hTim14/Pam18s-yTim16/Pam16s complex. Human Tim14/Pam18s and yeast Tim16/Pam16s were co-expressed in bacteria. Only the latter contained an octahistidine tag. Purified protein was analyzed using 16% SDS–PAGE, and stained with Coomassie blue. A) Ni-agarose column. B) Cleavage of the octahistidine tag with TEV protease. C) Gel filtration column. The fraction number is indicated on top of the gel. M: molecular weight markers (a similar approach has been used to isolate the yeast Tim14/Pam18-Tim16/Pam18 complex [26]).

It was demonstrated that in solubilized mitochondria yeast Tim14/Pam18 and Tim16/Pam18 form a stable hetero-oligomeric complex with a reduced ability to stimulate the ATPase activity of mtHsp70, compared to Tim14/Pam18 alone. Additionally, the formation of the Tim14/Pam18-Tim16/Pam16 complex is essential for the correct function of both proteins *in vivo* [16]. Previous studies have also shown that the J-domains alone of Pam18/Tim14 and Pam16/Tim16 are able to form a complex [16, 20,26]. Therefore, in order to test the ability of human Tim14/Pam18 to interact with yeast Tim16/Pam16 and to form a complex, we cloned the soluble J domains of both proteins (a.a 24–116 and a.a 25–130, respectively) and co-expressed them in bacteria. Since only the yeast Tim16/Pam16s (a.a 25–130 of Tim16/Pam16) contains an octahistidine tag, the efficient purification of both proteins during all steps of isolation indicates that a complex is indeed formed between hTim14/Pam18s and yeast yTim16/Pam16s (figure 1). The proof of concept for this strategy, using the homologous yeast proteins, was published previously [26]. As shown in Figure 1, human Tim14/Pam18 was able to form a complex with yeast Tim16/Pam16. Using the same strategy, we were also able to demonstrate complex formation between yeast Tim14/Pam18 and human Tim16/Pam16 (not shown) and between human Tim14/Pam18 and human Tim16/Pam16 (not shown). We conclude that the putative human Tim14/Pam18 and Tim16/Pam16 do interact, *in vitro*, to form heter-oligomers and that they can also form heterologous complexes with their yeast counterparts.

### 2.2. The oligomeric state of recombinant Tim14/Pam18s-Tim16/Tim16s complexes

**Figure 2.**
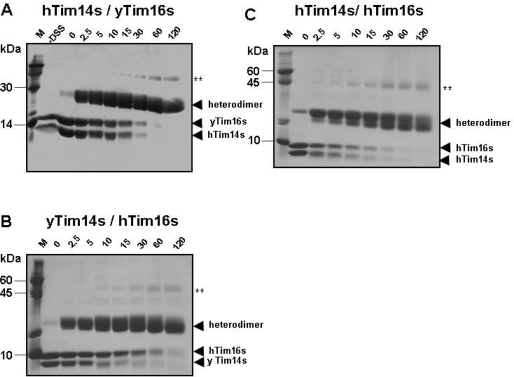
Cross-linking experiments of the different Tim14/Pam18s-Tim16/Pam16s complexes. A) Cross-linking of the hTim14/Pam18s-yTim16/Pam16 complex. B) Cross-linking of the yTim14/Pam18s-hTim16/Pam16s complex. C) Cross-linking products of the hTim14/Pam18s-hTim16/Pam16s complex. Cross-linking was carried out with 1 mM DSS at room temperature in a buffer containing 20 mM Na-Hepes pH 7.4, 200 mM NaCl, 100 mM KCl and 1 mM MgCl_2_, at a protein concentration of 15 μM. The cross-linking reactions were stopped at different times by addition of 10 μl SDS sample buffer and further boiling for 5 minutes. The cross-linking products (15 μl) were analyzed using 16% acrylamide gels. **minor amount of higher oligomeric cross-linked forms, presumably tetramers, of Tim14/Pam18s-Tim16/Pam16s. M: molecular weight markers. The Mw is indicated to the left of the gel.

Previous studies showed that yTim14/Pam18s and yTim16/Pam16s assemble into hetero-dimers in solution, [16,19,26]. We used cross-linking to examine the oligomeric state of the three complexes purified in this study (one homologous human hetero-dimer and two heterologous human/yeast hetero-dimers). The purified complexes were cross-linked using DSS and the cross-linking products were analyzed using SDS-PAGE. The results presented in figure 2 show clearly that upon exposure to the cross-linker, bands representing the monomeric proteins weaken while at the same time hetero-dimer cross-linking products appear, in all three complexes examined. A similar pattern, indicative of dimeric molecules, was observed previously for the homologous yeast complex [26]. The similarity in the cross-linking pattern of the three complexes examined in this study, together with the previously described cross-linking pattern for the yeast Tim14/Pam18-Tim16/Pam16 complex, supports the idea that the interaction between the yeast and human Tim14/Pam18-Tim16/Pam16 complex is evolutionarily conserved. This suggests that their function is also most likely conserved.

### 2.3. The folding and thermal stability of the purified complexes

**Figure 3.**
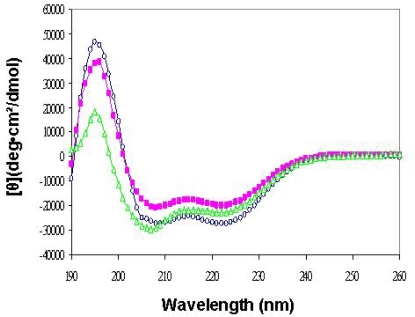
Represenatative Circular Dichroism (CD) analysis of some of the purified proteins. Spectra were obtained at 4° C in PBS buffer (pH=7.4). ( 
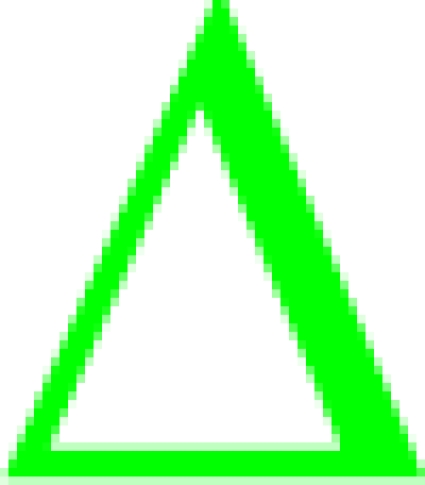
) CD spectra of hTim16/Pam16s ( 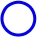) CD spectra of yTim14/Pam18s-hTim16/Pam16s complex ( 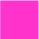) CD spectra of hTim14/Pam18s/-hTim16/Pam16s complex. Similar spectra were obtained for all constructs presented in Table 1.

Yeast Tim14/Pam18 and Tim16/Pam16 are members of the J and J-like protein families, respectively. These proteins are characterized by a large domain composed of three α helices. As such, they exhibit distinct CD spectra [26]. Since hTim14/Pam18 and hTim16s/Pam16 are homologues of these yeast proteins, oligomers containing them should display a similar CD spectrum, assuming they fold into similar structures. To determine whether this is indeed the case, we carried out a CD analysis to determine the secondary structure of the purified constructs of hTim14/Pam18s (not shown), hTim16/Pam16s (figure 3), a complex of yTim14/Pam18s-hTim16/Pam16s (figure 3) and a complex of hTim14/Pam18s-hTim16/Pam16s (figure 3). A similar CD spectrum was obtained for all proteins tested, which was characterized by two minima at 222 nm and at 208 nm, typical of α-helical proteins (Figure 3). The results indicate that the human and yeast purified proteins are similarly folded and contain essentially α-helical structures.

We reported previously that yTim14/Pam18s and yTim16/Pam16s are only marginally stable proteins that undergo unfolding at very low temperatures (Tm values for the individual proteins of 16.5°C and 29°C, respectively) [26]. Upon mixing the purified proteins, or when both proteins are co-expressed in bacteria, yTim14/Pam18s and yTim16/Pam16s form a hetero-dimer that is thermally more stable (Tm of ~40° C) compared to the individual proteins. Consequently, we proposed that any dissociation of the yeast Tim14/Pam18-Tim16/Pam16 hetero-dimer complex *in vivo* would theoretically lead to denaturation of these essential import components. Therefore, it was speculated that the formation of a stable Tim14/Pam18-Tim16/Pam16 complex is favored *in vivo* and the regulation of their function on the translocation motor is exerted through conformational changes [26].

We determined unfolding midpoints (Tm) by monitoring changes in the secondary structure content, detected by CD spectroscopy at 222 nm. The unfolding midpoints of individual Tim/Pam proteins and their complexes are summarized in Table 1. The results indicate that while the three proteins yTim14/Pam18, yTim16/Pam16s and hTim16/Pam16s are essentially unstable proteins (Tm of 16.5, 29, 22.5° C, respectively) the human Tim14/Pam18s is significantly much more stable (Tm of ~45). Complex formation between the yeast proteins, yTim14/Pam18 and yTim16/Pam16s, and subsequent conformational changes lead to the stabilization of their complex [26]. Interestingly, when hTim16/Pam16s interacts with hTim14/Pam18s, the stability of the complex is similar to that of the latter protein. Thus, two different factors contribute to the stability of yeast and human Tim/Pam proteins. In the case of the yeast proteins, the individual proteins are significantly less stable than their complex. Thus, complex formation between them probably increases their folding which in its turn increases complex stability (41° C compared to 16.5° C and 29° C for the individual proteins). In the case of the human proteins, the Tm of their complex is very close to that of hTim14/Pam18s. In general, when we formed a complex that contained one of the human Tim/Pam proteins, the stability of the complex was not increased further than the stability of either of the individual proteins. We conclude that the thermal stability of human Tim14/Pam18s determines the stability of the full human complex.

### 2.4. The effect of the y Tim14/Pam18-hTim16/Pam16 complex on the ATPase activity of yeast mtHsp70

**Figure 5.**
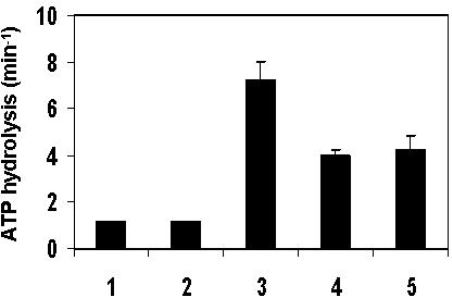
The effect of human Tim/Pam proteins on the ATPase activity of yeast mtHsp70. The ATPase assay was carried using the pyruvate kinase/lactate dehydrogenase-coupled assay as described previously [33]. The reaction mixture (total volume of 300 μl) contained the following components: 50 mM Tris-HCl pH=7.4, 50 mM K-Acetate pH=7.4, 10 mM Mg-Acetate pH=7.4, 0.3 mM NADH, 0.2 mM phosphoenolpyruvate, 20 units of pyruvate kinase, 10 units of lactate dehydrogenase and the indicated combinations of the following proteins mtHsp70 (2.5 μM), Mge1 (5 μM), full length Tim14/Pam18 and the soluble domain of Tim16/Pam16 from either *Saccharomyces cerevisiae* or human (2.5 μM). The reaction was initiated by the addition of 2 mM ATP. Rates were extracted from the linear phase of the reaction. Each column represents at least four independent repeats of the experiment. 1) mtHsp70. 2) mtHsp70+Mge1. 3) mtHsp70+Mge1+yTim14/Pam18 (full length). 4) mtHsp70+Mge1+yTim14/Pam18 (full length)+yTim16/Pam16s. 5) mtHsp70+Mge1+yTim14/Pam18 (full length)+hTim16/Pam16s.

As mentioned above, mtHsp70 serves as the core of the mitochondrial protein import motor [9,27,28]. This chaperone drives insertion of unfolded precursors into the matrix in an ATP-dependent manner. *In vivo*, mtHsp70 functions with the aid of several co-chaperones that regulate its ATPase activity. The first is the nucleotide exchange factor, Mge1, which enables ADP/ATP exchange and recycling of mtHsp70 [29–31]. The second co-chaperone is the yeast Tim14/Pam18, known to have a major role in stimulating the ATPase activity of mtHsp70 [11,14,15]. The third component, yeast Tim16/Pam16, antagonizes the function of Tim14/Pam18. Tim16/Pam16 specifically inhibits the Tim14/Pam18-induced ATPase stimulation of mtHsp70 [16,19]. We wanted to examine whether the human Tim/Pam homologues can affect the ATPase activity of yeast mtHsp70. An effect on ATP hydrolysis will indicate that not only the structure of the human Tim/Pam proteins is conserved, but also their function. To this end, we studied the ATPase activity of yeast mtHsp70 in the presence of the various co-chaperones (Figure 5).

In the absence of any additional components or in the presence of Mge1, mtHsp70 displays a low basal ATPase activity (1 turnover/min). Further addition of the purified yTim14/Pam18, containing a J-domain, significantly increases the rate of hydrolysis. Upon addition of the yTim16/Pam16s, there is an evident decrease in the ATPase activity [19,32], approximately 65% compared to the maximal hydrolysis activity, obtained for mtHsp70 in the presence of Mge1 and yTim14. These results demonstrate that the J domain of Tim14/Pam18 is less active in stimulating mtHsp70’s ATPase activity when in complex with the soluble domain of Tim16/Pam16. This is in agreement with previous reports [19,32]. A similar effect was observed using human Tim16/Pam16s, which inhibited the increase in the ATPase activity of mtHsp70 (obtained due to the presence of yTim14/Pam18) by almost 50%. The latter result indicates that hTim16/Pam16s can replace its yeast Tim16/Pam18s homologue*, in vitro,* and can act as a negative regulator of yTim14/Pam18. Based on the ATP hydrolysis experiments, we conclude that the function of Tim/Pam proteins is conserved between yeast and humans.

## 3. Experimental Section

### 3.1. Cloning and Purification of the proteins used in this study

Human Tim14/Pam18 and human Tim16/Pam16 were isolated by PCR from a human cDNA library and cloned as individual proteins or complexes into a modified version of the bacterial expression vector pET21d, in which a TEV protease site was inserted between the histidine tag and the N-terminus of the protein. The proteins used in this study are detailed in Table 2. The purification of the constructs 1–2 in Table 2 was carried out as described previously for the soluble domains (constructs 3–4) of yeast Tim14s/Pam18s and Tim16s/Pam16s [26]. Constructs 5–7 were purified as described previously for construct 8 [26]. Constructs 9–10 were purified carrying an octa-histidine tag on Ni-agarose following manufacture's protocol. In constructs 1–8, the hisitidine tag was removed from the final purified protein by proteolysis with TEV protease. In constructs 9–10, the hisitidine tag was not removed from the final purified protein.

### 3.2. Circular dichroism (CD)

All CD measurements were performed with an Aviv CD spectrometer, as described previously [26].

### 3.3. Cross-linking experiments

Cross-linking of the proteins was carried out at room temperature in 20 mM of Na-HEPES (pH 7.4), containing 100 mM of KCl, with 1 mM DSS. A protein concentration of 0.5 mg/mL was used. The cross-linking reaction was stopped at different times by the addition of SDS-containing sample buffer and boiling for 5 min. The cross-linking products (20 μL) were analyzed by 16% SDS-PAGE [26].

### 3.4. Miscellaneous

mtHsp70 was purified as described in [34]. ATPase assays were carried out as described in [33]. The concentrations of proteins were determined with the Bicinchoninic Acid Protein Assay (Sigma; Cat. no.B9643) using BSA as a standard [33].

## 4. Conclusions

The major goal of this study was to characterize the structure of human Tim14/Pam18 and Tim16/Pam16 (individually and in complex) and to show that their function is evolutionarily conserved with their yeast homologues. Human Tim14/Pam18 and Tim16/Pam16 (originally known as DNAJC19 and Magmas, respectively), have recently been identified [21,22,25]. So far, very limited *in vitro* data has been accumulated regarding these two proteins. The results presented in this study show clearly that the human proteins associate and form a complex *in vitro*. The formed complex has similar properties to those of a complex formed by yeast Tim14/Pam18 and Tim16/Pam16: i). Both complexes assemble into hetero-dimers containing one copy of each subunit. ii) Both complexes exhibit CD spectra typical of proteins with α helical structures. Interestingly, we found that factors contributing to the stability of the yeast complexes against heat denaturation are distinct from those that stabilize the human proteins. In the case of the yeast complex, stabilization is achieved by simultaneous conformational changes on both proteins, Tim14/Pam18 and Tim16/Pam16. In contrast, stabilization of the folded state of the human complex is governed by the human Tim14/Pam18 protein, which is more stable to heat denaturation than the other individual proteins. The conservation of the structural properties between the yeast and human proteins (including their ability to form heterologous complexes) together with the ability of human Tim16/Pam16 to affect the ATPase activity of the yeast mitochondrial Hsp70 chaperone suggest that the function of both complexes is evolutionary conserved from yeast to humans.

## Figures and Tables

**Figure 4.**
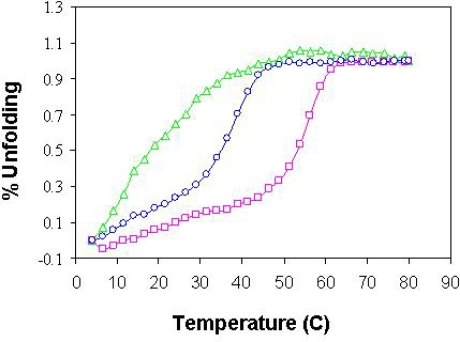
Thermal denaturation of purified proteins and complexes, as obtained from CD spectroscopy: Representative experiments. Similar experiments were carried out to extract the data presented in table 1. The fraction of denatured protein (% unfolding) was obtained by following changes in the ellipticity at 222 nm at various temperatures. A) ( 
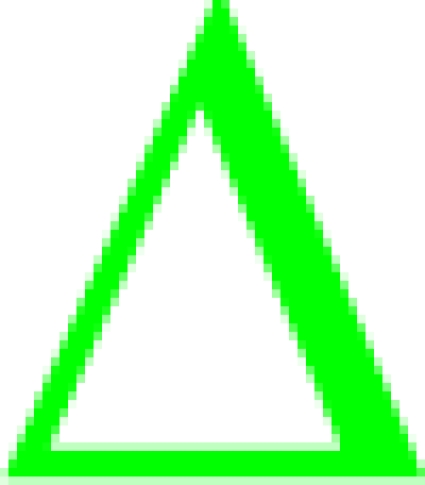
) 0.1 mg/mL hTim16/Pam16s. B) ( 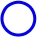) 0.1 mg/mL yTim14/Pam16s-hTim16/Pam16s. C) ( 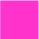) 0.1 mg/mL hTim14/Pam18-hTim16/Pam16s.

**Table 1.** Tm values of Tim14/Pam18 and Tim16/Pam16 constructs. Tm values were extracted from experiments carried out as described in figure 4.

**Name of construct**	**Tm (**°**C)**
yTim14/Pam18s	16.5
yTim16/Pam16s	29
yTim14/Pam18-yTim16/Pam16	41
hTim14/Pam18s	45
hTim16/Pam16	22.5
hTim14/Pam18s-yTim16/Pam16s	52
yTim14/Pam18s-hTim16/Pam16s	35
hTim14/Pam18s-hTim16/Pam18s	49

**Table 2.** Constructs used in this study and their abbreviations.

	**Description**	**Amino acids included in construct**	**Abbreviated name**

1	soluble domain of human Tim14/Pam18	24–116	hTim14s/Pam18s
2	soluble domain of human Tim16/Pam16	24–125	hTim16s**/**Pam16s
3	soluble domain of yeast Tim14/Pam18*	84–168	yTim14s
4	soluble domain of yeast Tim16/Pam16*	25–130	yTim16s/Pam16s
5	yeast Tim14s/Pam18s in complex with human Tim16s/Pam16s	84–168	yTim14s/Pam18s-hTim16s/Pam16s
		24–125	
6	Human Tim14s/Pam18s in complex with yeast Tim16s/Pam16s	24–116	hTim14s/Pam18s-yTim16s/Pam16s
		25–130	
7	human Tim14/Pam18 in complex with human Tim16s/Pam16s*	24–116	hTim14s/Pam18s-hTim16s/Pam16s
		24–125	
8	yeast Tim14s/Pam18s in complex with yeast Tim16s/Pam16	84–168	yTim14s/Pam18s-yTim16s/Pam16s
		25–130	
9	full length yeast Tim14	1–168	yTim14/Pam18
10	yeast Tim14/Pam18 in complex with yeast Tim16/Pam16 (both full length)	1–168	yTim14s/Pam18s-yTim16s/Pam16s
		1–149	

* The purification of these constructs was reported previously.
